# Functions and Clinical Applications of Exosomes in Gastric Cancer

**DOI:** 10.7150/ijbs.98087

**Published:** 2025-02-28

**Authors:** Zhi Zheng, Yuhao Zhai, Xiaosheng Yan, Zimeng Wang, Haiqiao Zhang, Rui Xu, Xiaoye Liu, Jun Cai, Zhongtao Zhang, Yuxi Shang, Jun Zhang, Jie Yin

**Affiliations:** 1 Department of General Surgery, Beijing Friendship Hospital, Capital Medical University, Beijing, China.; 2 Beijing Key Laboratory of Cancer Invasion and Metastasis Research, Beijing, China.; 3 National Clinical Research Center for Digestive Diseases, Beijing, China.; 4 Beijing Institute of Clinical Medicine, Beijing, China.; 5 Department of Pathology, Beijing Friendship Hospital, Capital Medical University, Beijing, China.; 6 Department of Hematology, Fuxing Hospital, Eighth Clinical Medical College, Capital Medical University, Beijing, China.

**Keywords:** gastric cancer, exosomes, mechanism, clinical application, advancement

## Abstract

Gastric cancer is a common and highly invasive type of malignant tumor, the pathogenesis of which remains unclarified. However, exosomes are now known to play important roles in gastric cancer development and treatment. Cells use exosomes for the packaging and transportation of a variety of bioactive molecules, such as proteins, double-stranded DNA, and micro-ribonucleic acids, to other sites. Exosome-specific membrane structures and exosomal contents are widely involved in processes that facilitate material exchange and intercellular communication between gastric cancer cells. They help in forming a pre-metastatic microenvironment, promoting the proliferation and apoptosis of gastric cancer cells, and driving invasion, metastasis, and resistance to anti-tumor drugs. In this review, we aimed to summarize the findings of research articles indexed in the PubMed, Web of Science, and Embase databases and published up to May 31, 2024, on the role of exosomes in the pathogenesis of gastric cancer and their potential clinical applications in its treatment. Thus, research on exosomes may lead to breakthroughs in the early diagnosis of gastric cancer and identification of novel treatments.

## Introduction

Gastric cancer (GC) is the fifth most common type of cancer worldwide, with the third highest mortality rate among all types of malignant tumors; therefore, it poses a serious threat to human health 1. In 2019, the incidence rate of GC in China was 43.1/100,000 people, the second highest among all malignant tumor types, with a mortality rate of 29.6/100,000 individuals 2. Although strategies for the prevention and treatment of GC are typically aggressive, the main factor affecting the prognosis of patients with the disease is tumor metastasis. This is driven by multiple intrinsic and extrinsic molecular signaling cascades within both tumor and stromal cells. The local spread and distant metastasis of GC occur through invasion or the secretion of related factors 3. Although advances in surgery, chemotherapy, and radiotherapy have improved the prognosis of patients with advanced GC, the survival outcomes remain poor, with a 5-year survival rate of only 10-30% 4. In the early stages, patients with GC are usually asymptomatic, which can lead to a delayed diagnosis and missed opportunities for radical surgical intervention. Therefore, early identification is crucial for improving survival rates among patients with GC who are able to undergo complete surgical resection of the lesion. Moreover, novel noninvasive biomarkers that exhibit high sensitivity and specificity must be identified for early-stage GC 3.

Exosomes have been widely used in the diagnosis and treatment of tumors and have become a hot topic in recent years. Exosomes play an important role in tumor invasion, metastasis, and invasion, which has not only been studied in solid tumors, such as ovarian and breast cancer, but also as a general law of tumor occurrence and development. Because of this, exosomes can be used as an important tool for drug or antagonistic drug resistance in the treatment of many types of tumors. GC also involves exosomes in the process of disease development and treatment; however, it is still different from other tumors not only in the relevant pathways but also in clinical applications.

Over the past decade, there has been growing research interest in determining whether exosomes can be used as diagnostic or treatment-related biomarkers 5. This is because they are known to play important roles not only in tumorigenesis, invasion, and metastasis but also in the mechanisms that promote immune escape or drug resistance 3, 6-10. In this review, we aimed to highlight and discuss the known and potential relationships between exosomes and the pathogenesis and treatment of GC, including the mechanisms of action and possible clinical applications.

## 2. Methods

A literature search was conducted to identify articles related to GC that were published up to May 31, 2024, and indexed in PubMed, Web of Science, and Embase. The following search terms: ((gastric cancer) OR (adenocarcinoma of esophagogastric junction)) AND ((extracellular vesicles) OR (exosomes)) AND ((epidemiology and etiology) OR (biological characteristics) OR (regulation mechanism) OR (clinical applications)) were used. Further searches and analyses were performed using various combinations of the aforementioned search terms. The retrieved articles were further scrutinized to identify additional relevant articles from their references. Qualitative and quantitative data were extracted via cyclical interpretation of each article to avoid overlooking potentially valuable data. A total of 218 articles were retrieved.

## 3. Epidemiology and etiology of GC

GC is a common type of malignant tumor that exerts considerable deleterious effects on human health worldwide 11. The causative factors of GC are diverse; however, its etiology is known to be related to infectious, dietary, and genetic factors 12-14. For example, *Helicobacter pylori* infection is an important pathogenic agent that is closely associated with the occurrence and progression of GC 15. It has been classified as a Group I carcinogen, and studies have shown that people infected with this type of bacteria are three times more likely to develop non-cardiac cancers than are individuals without such infection 16. A meta-analysis showed that the incidence of *H. pylori* infection-induced GC was higher in Chinese, Japanese, and South Korean populations than in European and American populations, with the former populations experiencing significantly higher mortality rates than the latter 17. *H. pylori* infection, in combination with other risk factors, such as smoking, increases the risk of GC development 18, 19. Infection with the Epstein-Barr virus (EBV) has also been identified as a predisposing factor for GC, and these patients are more likely to experience GC metastasis than are non-infected patients 20. Moreover, the incidence of post-surgical gastric stump cancer is four times higher in patients with such infections 21. The incidence of GC associated with viral infection has been shown to involve exosomes, which act as communication agents that can contain viral genetic material, miRNAs, and proteins, such as EBV latent membrane protein 1, which are delivered to the recipient cells 22. In addition to smoking, other unhealthy lifestyle factors, including drinking and obesity, increase the risk of GC 23. For example, some studies have found that high nitrite intake and excessive tea consumption are associated with the occurrence of GC 24, 25. This unhealthy lifestyle-induced GC is also affected by exosomes, and some studies have shown that exosomal circ0000670 promotes the development of cigarette smoke-induced GC, which also provides ideas for follow-up research. In addition, various intrinsic factors within the organism, such as LncRNA CCAT2, also influence the occurrence and development of gastric cancer 26-29.

In terms of genetic factors, approximately 10% of patients with GC have familial aggregation, and 1-3% of cases are hereditary 30; therefore, more attention should be paid to patients with a family history of the disease. Furthermore, early cancer screening should be conducted. However, owing to economic factors, early cancer screening is performed significantly less frequently in low- and middle-income countries than in advanced countries; this leads to differences between these countries in the proportion of GC cases detected in the early stages 11. In recent years, progress in research has significantly reduced the incidence and mortality of GC, especially in countries with historically elevated incidence rates, such as Japan and South Korea. However, Asian countries still have the highest GC incidence, collectively accounting for more than 70% of all new cases diagnosed globally in 2020, with East Asia exhibiting an age-standardized incidence rate as high as 22.4/100,000 individuals 31.

In addition to infectious, lifestyle, and genetic factors, sex is also known to affect the occurrence and development of GC, with an approximately 2-3 times higher incidence in men than in women. These findings suggest that men should be particularly attentive to gastric symptoms and should be encouraged to undergo early cancer screening 32.

Currently, the preferred treatment option for GC is surgical intervention, supplemented with neoadjuvant therapy and postoperative adjuvant chemotherapy 33. Patient prognosis is closely related to whether radical surgery can be performed; therefore, early detection, diagnosis, and treatment are important factors that affect clinical outcomes in patients with GC.

## 4. Introduction to the functions of extracellular vesicles

**Figure 1:**
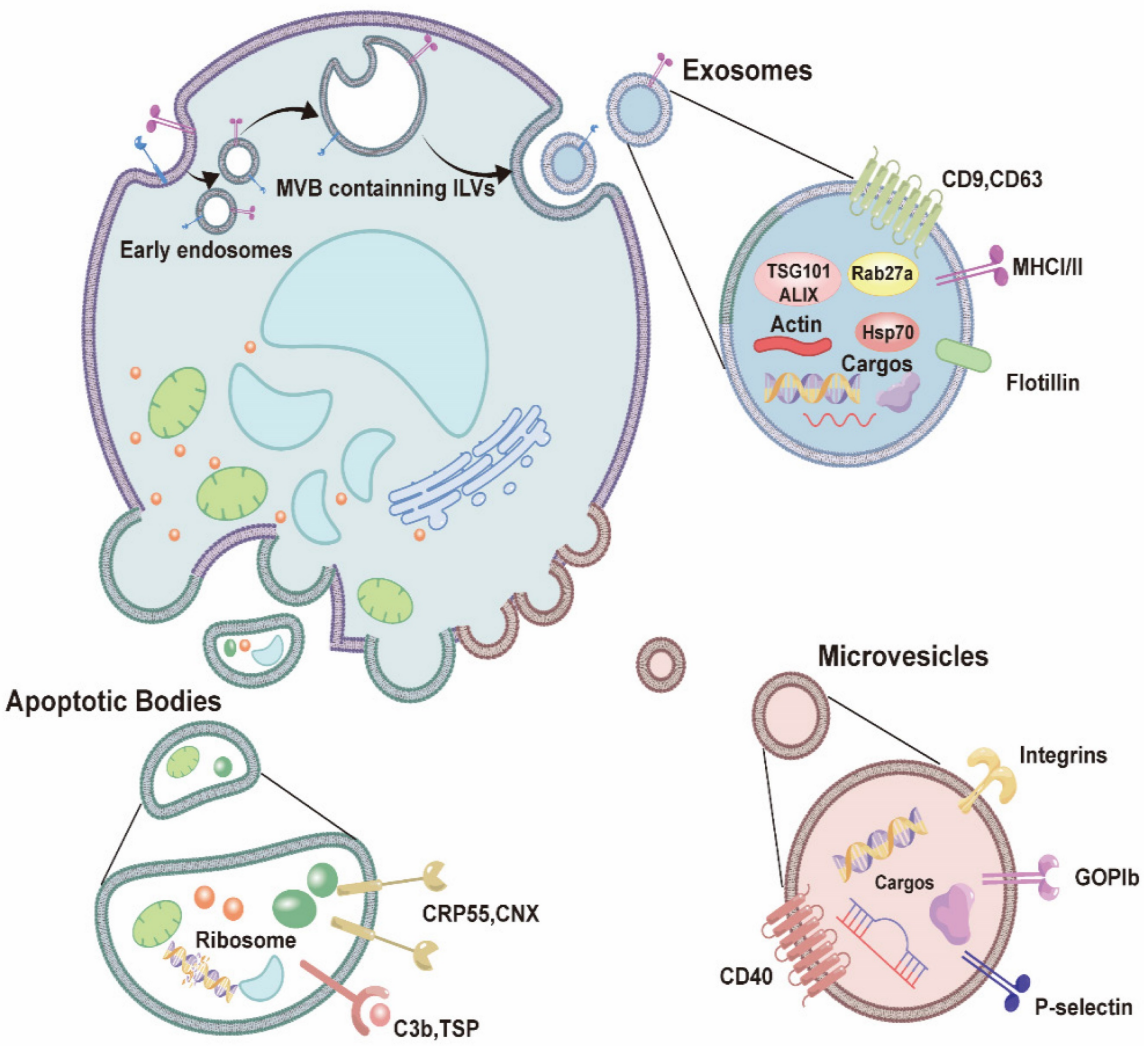
** The characteristic of extracellular vesicles.** Extracellular vesicles include apoptotic bodies, microvesicles, and exosomes. Among them, the biological mechanism of exosome production is that intraluminal vesicles (ILV) are generated within the cell sorting system and subsequently become enclosed within a multivesicular body (MVB), thereafter, transportation, anchoring, and fusion of the MVB with the cell membrane takes place, and the small exosomal vesicles are released extracellularly.

Extracellular vesicles have distinct membrane structures and are produced by and released from cells. Based on differences in their mechanisms of production, as well as their size and biophysical properties, extracellular vesicles can be further classified as apoptotic bodies (100-5000 nm), microvesicles (50-1000 nm), and exosomes (30-150 nm) 34. A special biogenetic mechanism leads to the production of exosomes: first, an intraluminal vesicle (ILV) containing specific contents is generated within the cell sorting system and will subsequently become enclosed within a multivesicular body (MVB). Following transportation, anchoring, and fusion of the MVB within the cell membrane, the small exosomal vesicles are released extracellularly 35** (Figure 1)**. These exosomes can carry a variety of substances, including nucleic acids, lipids, and proteins, allowing for the transportation of signaling molecules and communication between other cells and tissues 34, 36. Thus, exosomes play an important role in maintaining normal cellular homeostasis 37-39.

### 4.1 Biological characteristics of exosomes

Exosomes are nanovesicles that are released from cells into various bodily fluids, and the diameter of an MVB can range from 30 to 150 nm 30, 32. Exosomes exhibit a “cup-shaped” structure, with a phospholipid bilayer that is similar to that of the cell membrane; however, their membranes express exosome-specific protein markers, such as CD9, CD63, heat shock protein (HSP) 70, and tumor susceptibility gene (TSG) 101 protein 34, 40 **(Figure 1)**.

Harding and Pan first observed the release of these vesicles following the fusion of MVBs with the plasma membrane in rat and sheep reticulocytes by visualizing anti-transferrin receptor antibody labeling in 1983 41, 42. In 1987, Johnstone was the first to coin the term “exosome” to describe these small extracellular vesicles, based on the fact that their vesicular contents were able to modulate the activity of other cells following endocytosis 43. Originally, the production and release of exosomes were originally thought to be a means of cellular excretion of debris and waste products. However, in 1996, Raposo et al. reported that exosomes derived from the B lymphocytes of humans and mice could induce antigen-specific major histocompatibility complex class II-restricted T cell responses 44. This finding confirmed that exosomes play important roles in the homeostatic maintenance of the extracellular microenvironment, as well as in antigen presentation, stimulation of T cell proliferation, and the induction of immune responses. In addition, in 2007, Valadi et al. discovered that exosomes contain mRNAs and miRNAs capable of regulating cellular processes, suggesting that they play crucial roles in intercellular communication 45. These discoveries have resulted in increased global research interest in the properties and functions of exosomes in various biological processes and disease states.

### 4.2 Regulatory mechanisms of exosome production

The unique intracellular processes through which exosomes are produced determine their compositional complexity and functional diversity 46. The production of extracellular vesicles mainly involves the formation of ILVs, transportation of MVBs to the cell surface, and fusion of MVBs with the cell membrane. These processes are predominantly mediated by Rab proteins, which are small guanosine triphosphatases (GTPases) that are members of the Ras superfamily, syntenin-1, TSG101, apoptosis-linked gene-2-interacting protein X (Alix), syndecan-1, endosomal sorting complex required for transport (ESCRT) machinery, phospholipids, tetramolecular cross-linked members, ceramides, sphingomyelinases, and soluble N-ethylmaleimide-sensitive factor attachment protein receptor 47, 48.

Exosomes can be categorized into different subgroups based on their size, and both large and small exosomes exhibit unique N-glycosylation, protein, lipid, DNA, and RNA profiles, as well as distinct biophysical properties, resulting in differential secretion processes 49. Exosome production usually involves either the ESCRT-dependent or ESCRT-independent exosome generation pathway, although alternative pathways have been identified.

## 5. Regulation of exosomes in GC

**Figure 2:**
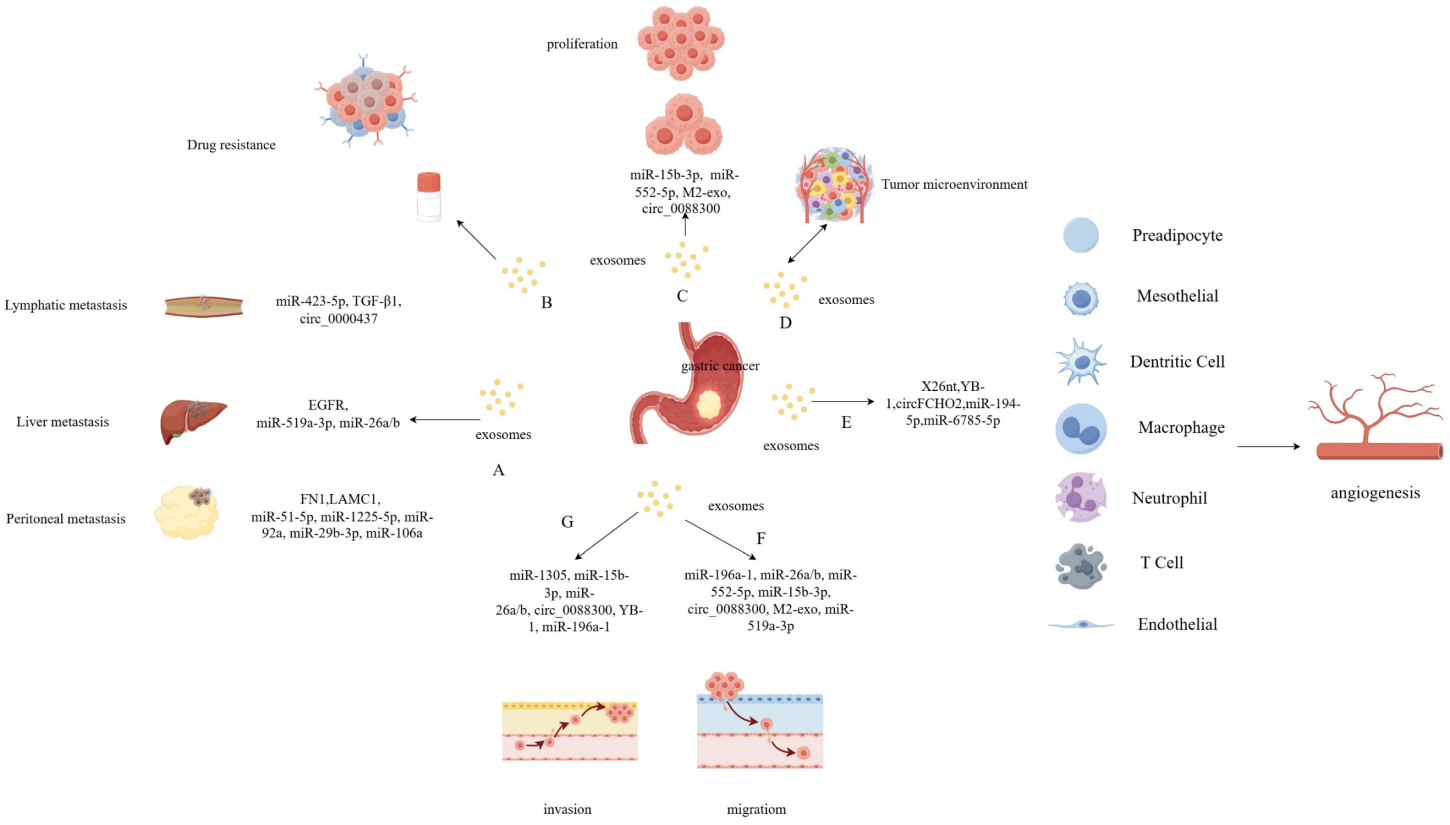
** Regulation and Function of exosomes in gastric cancer (GC).** Exosomes carrying certain contents are closely associated with GC proliferation and metastasis which include lymphatic-, peritoneal-, and liver-specific metastasis **(A)**. The extracellular vesicles derived from GC play an important role in drug resistance **(B)**, proliferation **(C)** and tumor microenvironment **(D)**. The gastric cancer cell derived exosomes play an important role in various processes related to tumor development, including angiogenesis **(E)**, migration **(F)**, and invasion **(G)**.

Exosomes are widely distributed within the tumor microenvironment, participating in intercellular interactions 50. The tumor microenvironment plays an important role in various processes related to tumor development, including angiogenesis, proliferation, migration, invasion, and drug resistance **(Figure 2)** 51. Recently, there has been growing research interest in the role of exosomes in GC, and the specific molecular mechanisms in these processes are diverse.

### 5.1 Exosomes and angiogenesis in GC

Excessive angiogenesis or abnormal remodeling can promote the progression of many diseases, including various cancers. Vascularization increases the likelihood of tumor cell metastasis, and angiogenesis is a key factor that drives tumor progression. Chen et al. reported that exosomes secreted by GC cells that carry the noncoding RNA X26nt can promote angiogenesis in human umbilical vein endothelial cells by reducing cadherin expression 52. Another study found that GC cell-derived exosomes that carry Y-box binding protein 1 can promote angiogenesis by increasing the expression of certain angiogenic factors 53. Zhang et al. reported that FCH and Mu domain containing endocytic adaptor 2 circular RNA (circ) can activate the Janus kinase 1/signal transducer and activator of transcription 3 pathway by acting as a sponge for miR-194-5p and promoting angiogenesis in GC 54. Chen et al. found that miR-6785-5p carried by exosomes from human umbilical cord mesenchymal hepatocytes were capable of inhibiting angiogenesis in GC 55. In summary, exosomes play an important role in modulating angiogenesis in GC, and exosomes derived from different cellular sources exert different effects.

### 5.2 Exosomes and tumor cell proliferation and migration in GC

Malignant tumors are characterized by abnormal proliferation, invasion, and metastasis, and all of these processes are closely associated with the effects of exosomes. One study found that exosomes from GC cells could induce the transformation and fibrotic carcinogenesis of surrounding non-cancerous gastric epithelial cells 56. Meanwhile, circ_0088300-containing exosomes derived from tumor-associated fibroblasts were capable of promoting the proliferation, migration, and invasion of GC cells by acting as a “sponge” for miR-1305 57. Exosomes derived from tumor-associated macrophages have also been shown to promote the proliferation and migration of GC cells by enhancing the phosphorylation of p38, increasing the expression of programmed death ligand 1, and inhibiting apoptosis 58. The inhibition of apoptosis is one of the mechanisms by which exosomes promote the progression of GC, and multiple studies have confirmed that miR-15b-3p- and miR-552-5p-containing exosomes derived from GC cells inhibit their apoptosis 59, 60. GC cell-derived exosomes can also promote the formation and progression of GC by increasing the likelihood of malignant transformation and inhibiting the apoptosis of surrounding normal cells. Exosomal delivery of molecules that can block these cellular processes could represent a new therapeutic direction for GC treatment.

In clinical practice, exosomal nucleic acids and proteins could serve as potential diagnostic and prognostic biomarkers for GC. By comparing the contents of cytoplasmic exosomes between patients with GC and healthy individuals, differentially expressed proteins and nucleic acids were identified, and their potential value as clinical biomarkers was evaluated. Proteins are also important functional units of exosomes. While most proteins in exosomes lack specificity, a certain number of them possess specific biological functions. Currently, validated proteins in research include TGF-β, Human gastrokine 1 (GKN1), apolipoprotein E (ApoE), among others 61-64. Most of these proteins are involved in the progression and metastasis of gastric cancer, and they can also promote gastric cancer growth by activating the Wnt/β-catenin pathway. The levels of UEGC1 long noncoding RNA (lncRNA) in the plasma exosomes of patients with early GC were shown to be higher than those in healthy individuals. In addition, its diagnostic performance was higher than that of carcinoembryonic antigen, suggesting that it could be used to effectively distinguish between individuals with and without precancerous lesions, such as those with atrophic gastritis 65. Analysis of the miRNA expression profiles of GC exosomes revealed that miR-1246 expression was significantly decreased, especially in patients with early GC and healthy individuals. Some studies have shown that miR-1246 can be used as an inhibitor of GC, and an increase in its expression levels can be indicative of an improvement in disease severity 66.

Because of the bilayer structure of exosomes, their contents are well-protected and do not readily undergo degradation, making them excellent tools to facilitate intercellular communication and serve as indicators of information transfer between GC and other cells through the general circulation. Exosomes will continue to be a hotbed of research related to the identification of underlying molecular mechanisms and the discovery of novel biomarkers of GC.

### 5.3 Exosomes and metastasis in GC

GC is often accompanied by peripheral lymph node metastasis. In 2009, Qu et al. first investigated the mechanisms through which exosomes modulate GC, revealing that cellular proliferation was induced through the activation of the phosphoinositide 3-kinase/Akt and mitogen-activated protein kinase/extracellular signal-regulated kinase (ERK) pathways 61. Moreover, it was shown that miR-217 and the let-7 miRNA family can promote GC cell proliferation and metastasis 61, 67-69. Another study by Shen et al. found that GC-derived exosomes contain cir_0000437, which induces lymph node metastasis through the HSPA2/ERK signaling pathway 70. LncRNA of the zinc finger nuclear transcription factor, X-box binding 1-type containing 1 antisense RNA 1, was shown to be overexpressed in both GC tissues and serum exosomes 71. Furthermore, GC exosomes have been shown to promote the adhesion of GC cells to related tissues by increasing the expression levels of intracellular fibronectin and laminin, thereby promoting metastasis. GC-derived exosomes also have an impact on the tumor microenvironment, with one study reporting that they were capable of stimulating macrophages to induce local inflammatory reactions through the activation of the nuclear factor kappa B (NF-κB) pathway, thereby promoting GC development 72. These exosomes were also capable of activating the transforming growth factor (TGF) β/Smad pathway in umbilical cord-derived mesenchymal stem cells by transporting TGF-β to the cells and promoting their conversion to cancer-associated fibroblasts to promote GC metastasis 73. Finally, exosomal miR-423-5p was shown to promote GC growth and metastasis by targeting a suppressor of fused homolog protein, exhibiting potential as a GC biomarker 74.

Celiac implantation metastasis is a common form of GC metastasis that is associated with a poor prognosis. Methods for predicting disease recurrence and implantation metastasis within abdominal tissues have historically included regular computed tomography, gastroscopy, and serum tumor marker quantification. However, in recent years, the measurement of exosomal contents to predict disease progression in patients with GC has gradually attracted attention. For example, Tokuhisa et al. reported that the presence of exosomal miR-21 and miR-1225-5p in peritoneal lavage fluid could be used as a novel biomarker of peritoneal metastasis following gastrectomy for GC, facilitating earlier diagnosis 75.

Internalization of tumor-derived exosomes by mesothelial cells has been shown to induce the expression of adhesion-related molecules, such as fibronectin 1 and laminin gamma 1. These can promote tumor-derived exosome adhesion to mesothelial cells in patients with GC, thereby promoting peritoneal metastasis. Ohzawa et al. demonstrated that patients with low expression levels of miR-29b-3p in exosomes collected via peritoneal lavage had a higher probability of experiencing peritoneal metastasis. These patients also exhibited worse overall survival outcomes than did those with high expression levels, suggesting that lower exosomal miR-29b-3p concentrations in peritoneal lavage samples could serve as a predictor of postoperative peritoneal metastasis 76. Additionally, Soeda et al. found that the levels of miR-21 and ex-miR-92a in plasma exosomes could be used as biomarkers to predict the occurrence of peritoneal metastasis and the prognosis of patients with stage II/III GC 77. Furthermore, Zhu et al. discovered that the presence of GC-derived exosomes containing miR-106a could promote the destruction of the mesothelial barrier via integration with peritoneal mesothelial cells, thereby promoting peritoneal metastasis of GC 78.

Collectively, these findings indicate that exosomes carrying certain contents are closely associated with GC metastasis, and the detection of such exosomes can help predict its occurrence.

### 5.4 Exosomes participate in the establishment of the pre-metastatic niche

Tumor metastasis is a complex, multistep process, and cancer cells do not diffuse randomly. Several studies have confirmed that the bioactive molecules carried by cancer cell-derived exosomes can be absorbed by the cells of specific organs, where they participate in the establishment of the pre-metastatic niche. Zhang et al. demonstrated that exosomes secreted by GC cells can regulate the microenvironment of the liver and “prepare” the way for liver metastasis 10. Patients with GC exhibit high serum levels of epidermal growth factor receptor (EGFR), which is transported from GC cells to the liver through exosomes, establishing a microenvironment that is conducive to liver-specific metastasis**.** By inhibiting miR-26a/b, EGFR activates the expression of hepatocyte growth factor, which is considered a tumor promoter 79, increasing the likelihood of liver metastasis in patients with GC and validating the “soil and seed” hypothesis of the tumor metastasis mechanism that is popular in clinical practice. Qiu et al. demonstrated that GC-derived exosomes transporting miR-519a-3p could mediate angiogenesis and promote liver metastasis by inducing the polarization of intrahepatic macrophages of the M2 phenotype 8.

Gastric, rectal, pancreatic, breast, and other cancers tend to metastasize within specific organs, such as the liver, lungs, and brain 80. Exosomal integrins fuse with target cells in a tissue-specific manner to initiate the establishment of a pre-metastatic niche that is conducive to organ-specific metastasis. Moreover, miRNAs contained in exosomes can enhance the permeability of endothelial cells and upregulate the expression of certain genes, leading to vascular remodeling and providing sufficient levels of nutrients to meet the metabolic demands of metastatic cancer cells 81.

In summary, exosomes participate in the formation of the pre-metastatic niche and play varying roles in different steps. These include metabolic reprogramming and immune and non-immune stromal recruitment, serving as messengers that connect the “seed” and “soil” and create a suitable microenvironment for tumor metastasis.

### 5.5 The signaling pathways of exosomes

Several studies have verified the relatively pivotal role of exosomes in the pathogenesis of GC 82, as well as their substantial impacts on processes such as invasion, metastasis, angiogenesis, immune evasion, and chemoresistance. In 2009, Qu et al. pioneeringly reported that extracellular vesicles (EVs) derived from GC cells contribute, at least partially, to tumor cell proliferation via the activation of the PI3K/Akt and MAPK/ERK signaling pathways. Furthermore, metabolic pathways involving the Cbl ubiquitin ligase family and Caspases are also implicated in this process 61, 83. In 2012, Gu and colleagues validated that the TGF-β/Smad signaling pathway, mediated by small extracellular vesicles (sEVs), triggers the transformation of umbilical cord mesenchymal stem cells into cancer-related fibroblasts. Furthermore, CD97 is believed to bolster the proliferation and invasive abilities of GC cells, and its role in promoting lymphatic metastasis in GC is correlated with the presence of EVs 84, 85. Moreover, sEVs derived from GC cells have the peritoneal capacity to induce the infiltration of peritoneal mesothelial cells (PMCs), which subsequently reinforces the subserosal invasion of tumors 86. In summary, the intricate interplay between cancer cells and PMCs expedites the invasion of the gastric wall and fosters metastasis. Furthermore, EVs occupy a crucial position in modulating the tumor microenvironment. For instance, sEVs originating from GC cells activate the phosphorylation of NF-κB in macrophages, thereby promoting cancer progression 72. Additionally, these sEVs trigger the production of programmed death receptor 1 (PD-1) positive tumor-associated macrophages, which facilitate tumor angiogenesis and metastasis 87. A comparable mechanism of action was documented by Shen et al. 88, where EVs were found to promote the polarization of N2 tumor-associated neutrophils, thereby inducing autophagy and augmenting tumor activation, ultimately facilitating the migration of GC 89. Ji et al. 90 and Wang et al. 91 have illuminated the critical function of EVs in mediating resistance to both 5-Fluorouracil (5-FU) and platinum-based treatments. These investigations are instrumental in uncovering and implementing potential alternative therapeutic agents that can effectively reverse drug resistance. Although research into EVs associated with GC remains in its nascent stages, there has been a notable improvement in both the quantity and quality of related studies lately. These advancements offer fresh perspectives on the mechanisms underlying the initiation and progression of GC.

### 5.6 Exosomes and drug resistance in GC

Drug resistance is a problem that must be overcome to optimize the treatment of various malignant tumors, including GC, which progresses rapidly and is associated with a poor prognosis. One study found that the main mechanisms of drug resistance included increased drug elimination, activation of compensatory mechanisms that repair DNA damage, apoptosis-related changes, enhanced drug metabolism, failure of targeted drug delivery, and epigenetic factors related to DNA methylation 92. In recent years, however, the role of exosomes in drug resistance in patients with GC has attracted attention. Wang et al. found that exosomal miR-155-5p secreted by paclitaxel-resistant GC cells could induce epithelial-mesenchymal transition and chemotherapeutic resistance in paclitaxel-sensitive GC cells by suppressing GATA binding protein 3 and tumor protein p53 inducible nuclear protein 1 expression 93. Another study showed that GC-derived exosomes containing circ_plasmacytoma variant translocation 1 could promote cisplatin resistance by regulating autophagy, invasion, and apoptosis in GC cells through modulation of the miR-30a-5p/yes-associated protein 1 axis 94. Exosome-derived miR-500a-3p has been shown to promote cisplatin resistance and the stemness of GC cells through the negative regulation of F-box/WD repeat-containing protein 7 95. Hypermethylation of transcription factor activator protein 2e has been shown to promote 5-fluorouracil resistance in GC cells through exosome-mediated delivery of miR-206a-5p and miR421 96. Meanwhile, some studies have shown that exosomes secreted by mesenchymal stem cells can mediate the resistance of GC cells to 5-fluorouracil by activating the Ca^2+^/Raf/mitogen-activated protein kinase/ERK signaling pathway while promoting the expression of multiple drug resistance-related proteins 90, 97.

Collectively, the findings of these studies suggest that exosomes are involved in mediating the chemotherapeutic resistance of GC cells. However, there has been little research on this topic to date, and exploration of the specific mechanisms driving these changes remains insufficient. Therefore, it is necessary to continue to explore the mechanism through which exosomes modulate drug resistance in GC to improve clinical outcomes.

### 5.7 Immunotherapy strategies for gastric cancer utilizing exosomes

EVs hold significant promise in the field of cancer immunotherapy 98. In clinical settings, the progression of PD-1/programmed death-ligand 1 (PD-L1) research has prompted the gradual initiation of immunotherapy-related clinical trials specifically targeting patients with MSI-H and dMMR. Recently, the field of tumor immunotherapy has garnered significant attention, particularly focusing on clinical research centered around inhibitors of PD-1 and PD-L1. The primary objective of these studies is to stimulate and enhance the activity of immune cells, thereby mitigating immune suppression and intensifying their tumor-killing capabilities 99. Previous research has predominantly centered its attention on the functional role of soluble PD-L1, with a comparatively limited exploration into the significance of EVs-PD-L1. Due to their secretory nature, sEVs are capable of both inhibiting and eliminating T cells within the local tumor microenvironment while also possessing the ability to traverse distant sites to perform additional functions. This dual-action mechanism could potentially serve as a potent facilitator of tumor immune escape 100. Fan et al. have documented that sEV-PDL1, owing to its stability and the disruption of T cell function triggered by Major Histocompatibility Complex-I expression, serves as an indicator of the immune status and holds potential in predicting the long-term prognosis of patients 101. Furthermore, research conducted by Zhang et al. in 2020 102 revealed that the administration of 5-FU may enhance the expression of EVs-PD-L1, potentially leading to immunosuppression in patients undergoing more than two cycles of chemotherapy. This finding has the potential to significantly influence the development of future comprehensive treatment plans for advanced GC, offering new insights into therapeutic approaches. Furthermore, it is anticipated that further intensive research will elucidate the intricate relationship between diverse drug therapies, ultimately enabling the formulation of precise and effective comprehensive treatment plans tailored to individual patients' needs.

The preponderance of related literature published within the last 3 years is likely a testament to the swift advancements in the fields of proteomics and transcriptomics. As these technologies continue to mature, it is widely believed that an increasing number of tumor-related effects attributed to the contents of EVs will be uncovered. Undoubtedly, the proportion of tumor-associated cargo within sEVs and the specific effects they elicit under the influence of multiple components remain unresolved questions that warrant further investigation.

## 6. The clinical application of exosomes in GC

Exosomes contain and transport a wide range of DNA, mRNA, and miRNA molecules, together with other genetic materials, as well as multiple classes of proteins and metabolites that can alter the tumor microenvironment and influence the development of drug resistance and disease progression in GC 103, 104. Monitoring exosome levels could allow for earlier tumor diagnosis and minimize the development of drug resistance. Therefore, exosomal targeting strategies have become research hotspots for GC diagnosis and treatment.

### 6.1 Exosomes as diagnostic biomarkers of GC

Genetic materials contained in exosomes, such as DNA and mRNA, can reflect the environmental status within an organism to a certain extent 105. Many studies have shown that exosomal miRNA levels significantly differ between patients with GC and healthy individuals. Therefore, they affect the occurrence and progression of tumors and highlight the potential value of exosomal miRNAs as predictive biomarkers of tumors 106. Because exosomes are widely distributed throughout the blood and other bodily fluids, including gastric juices, they can be used as simple and accessible noninvasive diagnostic and predictive biomarkers 107. The substances contained in exosomes are relatively stable and do not readily undergo degradation; therefore, their accuracy is higher than that of similar molecules within the plasma.

Several studies have been conducted on the use of exosomal miRNAs as important biomarkers of GC, including miR10b-5p, miR195-5p, miR20a-3p, and miR296-5p 108. Exosomal miR-101 was also found to be significantly downregulated during GC progression and metastasis, suggesting that it plays a protective role 109. Another study that monitored expression levels within exosomes in the plasma found that miR-23b could be used as an important marker to predict the likelihood of disease recurrence and clinical prognosis 110. In addition, the levels of certain exosomal miRNAs have been shown to be associated with survival times in patients with advanced GC. For example, the levels of miR-29 and miR-181 can predict the likelihood of peritoneal metastasis and are significantly associated with disease recurrence in GC 76, 111.

LncRNAs are also present in exosomes, and their levels can be monitored. The HOX transcript at the distal tip lncRNA can be used to diagnose tumors in their earlier stages, and its accuracy was shown to be higher than that of traditional biomarkers. Similarly, lnc1 levels can predict the progression of GC and were shown to be more accurate than commonly used markers, with multicenter studies confirming that it can be used to determine progression-free survival. Low expression levels can predict positive responses to adjuvant chemotherapy in patients with stage II/III GC 112. Other biomarkers, such as the solute carrier family 2 member 12-10:1 lncRNA have also been confirmed to be accurate indicators for the staging of GC and are associated with venous infiltration and lymph node metastasis 113.

Although few studies have been conducted to date, some have shown that exosomal circRNAs can also be detected in bodily fluids and may serve as GC biomarkers; for example, has_circ_0015286 was shown to be highly expressed in patients with GC, and the levels significantly decreased following surgical resection, suggesting this change could be indicative of a favorable prognosis 114. Patients with low exosomal hsa_circ_0015286 expression levels have also been shown to experience longer survival times and better prognoses. Ran guanosine triphosphatase-activating protein 1 circRNA may also serve as a potential diagnostic factor for GC, as its high expression levels in bodily fluids have been confirmed to be associated with later tumor/node/metastasis staging, lymph node metastasis, and poor long-term survival outcomes 115.

Exosomes also contain many specific and characteristic proteins and metabolites, some of which may be suitable diagnostic markers for GC. For example, PDL-1 is an important immunotherapeutic target, and high exosomal expression levels of the protein can predict a poor prognosis 101. A summary of the findings of studies related to the quantification of exosomal contents in GC is presented in **Table 1** 65, 67, 74, 101, 104, 114-132. We also summarize exosome-related proteins, and the relevant results are shown in **Table 2** 52, 85, 132-135.

Ultimately, there is ample evidence to suggest that exosomes and the molecules they transport could serve as diagnostic tools for GC, and there are several advantages to their use over traditional markers. First, exosomes can be isolated, and their contents can be quantified non-invasively, reducing pain and discomfort for patients. Second, exosomal miRNA monitoring can be used as a basis for the subsequent development of improved targeted therapies. Finally, secreted exosomes are homogeneous, and some of their contents could allow for the accurate determination of the activity occurring within tumor cells. However, further studies are required to determine the optimal biomarkers for GC.

Exosomes can also carry tsRNA in serum, and their levels can be used as biomarkers for the onset of gastric cancer. Plasma exosomal tRF-25, tRF-38, and tRF-18 serve as biomarkers for GC detection; tRF-25, tRF-38, and tRF-18 may also predict the prognosis of GC 136, 137.

The diagnostic role of exosomes in early gastric cancer is also crucial. Their functions are mainly manifested in two aspects. Firstly, they facilitate the acquisition of samples for gastric cancer diagnosis, with proven accuracy, which saves time in early cancer screening. The most commonly used samples are exosomes derived from serum, which can be used for tumor diagnosis. However, exosomes can also be obtained from other bodily fluids such as urine for diagnostic testing 138.

Secondly, they offer improved diagnostic accuracy in even earlier stages of gastric cancer. We expect that neuronal-derived exosomal miRNAs (Neu-Exo miRNAs) may serve as novel and precise biomarkers for gastric cancer diagnosis, and droplet digital PCR (ddPCR) could provide a promising tool for their detection 139.

Shao et al.'s research discovered that hsa_circ_0065149 is abnormally expressed in gastric cancer, with higher sensitivity and specificity than the widely used biomarkers CEA and CA19-9. The down-regulated expression of hsa_circ_0065149 serves as a prognostic predictor and a biomarker for early detection of gastric cancer. Non-coding RNAs from bone marrow stromal cell exosomes can be summarized and relevant features can be extracted through machine learning, which is of great significance in the diagnosis of early gastric cancer 140.

### 6.2 Exosomes in the regulation of resistance and drug carriers for pharmacotherapy

Exosomes are normally secreted by almost all cells, and their unique transportation mechanism makes them suitable for use as drug carriers. Exosomes are known to play a role in mediating chemotherapeutic resistance and in modulating the tumor microenvironment. They also have certain advantages as drug carriers due to the fact that their immunogenicity and cytotoxicity are low. The exosomal delivery of drugs and other substances can decrease the likelihood of drug resistance, as they are capable of more specific and efficient targeting compared with that of traditional solutes without undergoing degradation before being delivered to the target cells.

To date, most studies that have investigated the use of exosomes in GC have focused on the delivery of miRNAs or RNA, as well as the identification of polymorphisms that affect drug resistance in tumor cells 141, 142. For example, overexpression of miR-214 in GC cells has been shown to induce resistance to platinum-based chemotherapeutic agents and promote invasion and metastasis in patients with GC, both of which increase the likelihood of a poor prognosis. However, the underlying mechanisms through which these effects are mediated have yet to be elucidated. Some studies have shown that the exosomal delivery of anti-miR-214 compounds can significantly reduce resistance to platinum-based therapies, providing a novel treatment method for patients with cancer 91. Similarly, exosomal delivery of miR-501 can induce doxorubicin resistance by targeting BH3-like motif containing members of the B-cell lymphoma 2 family of proteins that regulate cellular apoptosis, thereby increasing the aggressiveness of tumor development and progression. One study showed that miR-501 knockdown could significantly reduce the establishment of a drug-resistant microenvironment 143. Another group showed that exosomal miR-21 can be directly transferred from macrophages to GC cells in order to confer chemotherapeutic resistance by inducing apoptosis and phosphorylating and activating proteins involved in the phosphoinositide 3-kinase/Akt pathway. Moreover, tumor cell apoptosis could be increased by inhibiting the transcription of miR-21 144. Exosomal miR-588 secreted by macrophages can also induce resistance to platinum-based therapies in patients with GC, making it a potential target for future GC interventions.

Owing to the large size of mRNAs, their transport and biological functions are often limited; however, the binding between exosomes and mRNAs can improve their stability and limit their immunogenicity 145. The exosomal transport of small interfering RNA to reduce the transcription of hepatocyte growth factor can inhibit the growth of GC cells and delay metastasis 146. In addition, the increased expression of antagonists that target exosomal delivery proteins can inhibit the function of CD37 on the surface of tumor cells while promoting their phagocytosis by macrophages and achieving adequate immune system regulation 147.

Exosomes can maintain the stability of membrane proteins during their transportation. Tumor necrosis factor-related apoptosis-inducing ligand, an exosomal surface protein, can transmit apoptotic signals to tumor cells and promote their apoptosis 148, and exosomal transportation of gastrokine-1 has been shown to be protective against the occurrence of GC 132.

The efficacy of trastuzumab emtansine in patients with human epidermal growth factor receptor-(HER) 2-positive GC is currently being studied, and it may become an important adjuvant and neoadjuvant drug for the treatment of patients with HER 2-positive GC. Some studies have shown that exosomes secreted by such tumors can bind and transport trastuzumab emtansine to the target tissues 149, inhibiting tumor growth and improving the prognosis of patients with GC. Despite these promising findings, research on the involvement of exosomes in the treatment of GC is still in its infancy, and the main difficulty lies in screening for and validating the use of protective exosomes for clinical applications.

In recent years, review articles on the functions of exosomes in gastric cancer have also been published. However, compared to other articles, this paper not only elucidates clinical applications but also explains the physiological functions exerted by exosomes, making its content more comprehensive 150, 151.

Although exosomes have many clinical applications in GC, there are still many limitations. At present, exosomes need efficient separation and purification technology in research and application. However, the current separation and purification cost is high, and the operation is complicated, which limits the wide application of exosomes to a certain extent. In addition, test samples often contain many exosome-like compositions, such as cell fragments and other extracellular vesicles or microvesicles, which can interfere with the accuracy of exosome identification. When exosomes are used as drugs for treatment, the limited capacity of exosomes to carry drugs may cause the concentration of drugs in target cells or target organs to be unable to meet the therapeutic demand, limiting their application in drug delivery. Therefore, relevant problems need to be solved in the follow-up research in order to provide help for clinical application.

In general, exosomes play an important role in the occurrence, development, diagnosis, and treatment of GC, which has both the universality of cancer and the uniqueness of GC.

## 7. Conclusion

In recent years, exosomes have become a research hotspot in the field of GC, with an increasing number of studies focusing on their unique roles and mechanisms of action. Exosomes have been confirmed to play important roles in the growth, proliferation, and metastasis of GC cells, and some may play vital roles as biomarkers for improving early diagnosis or in therapeutic strategies that minimize drug resistance or facilitate the targeted delivery of cargo as drug carriers.

Although the potential clinical applications of exosomes are obvious, four main challenges must still be overcome. First, the mechanisms of action of exosomes as key regulatory molecules in GC remain unclear, and the insufficient sample sizes of previous studies have hindered their clinical applications. Second, it remains unknown whether individual differences exist in the expression levels of certain exosomal markers for the early diagnosis of GC or whether such levels in a given patient vary in response to pharmacotherapy, infection, or other factors. Third, the accurate and efficient targeted delivery of exosomal contents must be further improved to optimize clinical outcomes. Finally, further studies are needed to investigate the effects of combination therapies involving exosomal carriers and traditional Chinese medicines, compounds, or monomers in patients with GC.

Overcoming the aforementioned challenges and further exploring the use of exosomes for GC diagnosis and treatment will take many years. However, continuous advances in science and technology and the increasing interest in this field of research are expected to usher in a new era, facilitating the earlier diagnosis of GC and improving patient prognosis in a clinical setting.

## Figures and Tables

**Table 1:** Exosomes as diagnostic biomarkers for GC

Biomarker type	Molecules	Exosome origin	Expression change	Description	Ref.
**miRNA**	miR-10b-5p, miR-101-3p, and miR-143-5p	Plasma	Upregulated	miR-10b-5p, miR-101-3p, and miR-143-5p identified as biomarkers of metastasis in GC.	117
	miR-4741, miR-32, miR-3149, and miR-6727	Tissues and plasma	miR-4741: upreguated;miR-32, miR-3149, and miR-6727: downregulated	Upregulated miR-32, miR-3149, and miR-6727 expression and downregulated miR-4741 expression lowered proliferative, migratory, and invasive capacities of cancer cells.	116
	miRNA-590-5p	Serum	Downregulated	Decreased exosomal miRNA-590-5p expression was related to the occurrence of GC and poor survival outcomes.	118
	miR-379-5p and miR-410-3p	Serum	Upregulated	miR-379-5p and miR-410-3p were significantly upregulated in metastasis.	119
	miR-181b-5p	Ascites	Upregulated	Upregulated miR-181b-5p was related to GC ascite formation.	111
	miR‑29s	Peritoneal lavage fluid or ascites	Downregulated	miR‑29s expression was downregulated in patients.	76
	miR-423-5p	Serum	Upregulated	Exosomal miR-423-5p can promote cancer growth.	74
	miR-217	Serum	Upregulated	miR-217 overexpression enhanced GC cell proliferation	67
	miR-1307-3p	Serum	Upregulated	Patients with GC metastasis had significantly higher expression levels of miR-1307-3p.	120
	miR-502-5p	Serum	Downregulated	Exosomal miR-502-5p acts as a suppressor in the development and progression of gastric cancer.	121
	miR-541-5p	Serum	Upregulated	miR-541-5p promotes GC cell progression	
**LncRNAs**	lncH19	Serum	Upregulated	LncH19 levels were significantly correlated with TNM staging.	122
	lncUEGC1	Plasma	Upregulated	LncUEGC1 expressed at significantly higher levels in patients with stage I GC.	123
	lncMIAT	Serum	Upregulated	Patients with gastric adenoma with elevated serum levels of exosomal MIAT were more prone to develop GC.	65
	lncGNAQ-6:1	Serum	Downregulated	Expression was significantly lower in the GC group.	124
	lncPCSK2-2:1	Serum	Downregulated	The expression level of exosomallncPCSK2-2:1 was correlated with tumor size, tumor stage, and venous invasion.	125
	lncCEBPA-AS1	Plasma	Upregulated	The AUC value of lncCEBPA-AS1 in discriminating patients with GC from healthy controls was 0.824.	126
	lncSLC2A12-10:1	Plasma	Upregulated	The expression level of exosomal lncSLC2A12-10:1 was correlated with tumor size, TNM staging, and lymph node metastasis.	127
	lncGC1	Plasma	Upregulated	Patients with low levels ofcirculating exosomal lncGC1 derived survival benefits from adjuvant chemotherapy.	113
	lncHOTTIP	Plasma and serum	Upregulated	Expression levels were typically upregulated in GC.	112
	lnc00152	Plasma	Downregulated	The sensitivity and specificity of plasma lnc00152 in the diagnosis of GC were 48.1% and 85.2%, respectively.	128
	lnc00853	Plasma	Upregulated	Expression levels were typically upregulated in GC.	129
**CircRNA**	hsa_circ_0015286	Tissue, plasma, and cancer cells	Upregulated	Result of the ROC curve analysis showed that AUC of hsa_circ_0015286 was 0.778.	130
	hsa_circ_0065149	Plasma	Downregulated	Levels were significantly decreased in patients with early GC.	114
	circ_RanGAP1	Plasma	Upregulated	High expression levels in bodily were confirmed to be associated with later TNM staging.	131
**Protein**	TRIM3	Serum	Downregulated	The expression levels of TRIM3 mRNA and protein were decreased in GC tissues.	115
	PD-L1	Blood exosome surface	Upregulated	Exosomal PD-L1 levels were an independent prognostic factor in GC.	104
	Tetraspanin 8	Plasma	Upregulated	Gastrokine 1 protein is a potential theragnostic target for GC.	101

Abbreviations: AUC, area under the curve; CEBPA, CCAAT/enhancer-binding protein alpha; circRNA, circular ribonucleic acid; GC, gastric cancer; GC1, glutamate carrier 1; GNAQ, guanine nucleotide-binding protein G(q) subunit alpha; HOTTIP, HOXA transcript at the distal tip; lncRNA, long noncoding ribonucleic acid; MIAT, myocardial infarction-associated transcript; miRNA, micro-ribonucleic acid; PCSK2, proprotein convertase 2; PD-L1, programmed death-ligand 1; RanGAP1, Ran guanosine triphosphatase-activating protein 1; ROC, receiver operating characteristic; SLC2A12, solute carrier family 2 member 12; TNM, tumor/node/metastasis; TRIM3, tripartite motif containing 3; UEGC1, up-regulated in the exosomes of gastric cancer 1.

**Table 2:** Exosomes related protein for GC

	Mechanism	Function	Ref.
DICER	miR-107 targets 3' UTRs of DICER and PTEN in MDSCs	DICER downregulation promotes MDSC expansion	133
YB-1	Up-regulation of angiogenic factors in endothelial cells	Promote gastric cancer angiogenesis	134
X26nt	Decrease vascular endothelial cadherin	Promote angiogenesis	52
GKN1	Tumor suppressor protein	Inhibit cell proliferation and induce cell apoptosis	132
TRIM3	TRIM3 knockdown regulates stem cell factors and EMT regulators	Promote the growth and metastasis of gastric cancer	135
CD97	Activate the MAPK signaling pathway	Promote cell proliferation and invasion	85

Abbreviations: YB-1, Y-box binding protein 1; X26nt, A 26-nt-long ncRNA; GKN1, Gastrokine 1; TRIM3, Tripartite motif-containing 3

## Abbreviations

Alix: apoptosis-linked gene-2-interacting protein X
circRNA: circular ribonucleic acid
EGFR: epidermal growth factor receptor
ERK: extracellular signal-regulated kinase
ESCRT: endosomal sorting complex required for transport
GC: gastric cancer
GTPases: guanosine triphosphatases
Hrs: hepatocyte growth factor-regulated tyrosine kinase substrate
HSP: heat shock protein
ILV: intraluminal vesicle
lncRNA: long noncoding ribonucleic acid
MSC: mesenchymal stem cell
CAF: cancer-associated fibroblast
EMT: epithelial mesenchymal transition
*H. pylori*: *Helicobacter pylori*
MBV: multivesicular body
miRISC: miRNA-induced silencing complex
miRNAs: micro-ribonucleic acids
mRNA: messenger ribonucleic acids
TSG: tumor susceptibility gene

## References

[1] Smyth EC, Nilsson M, Grabsch HI, van Grieken NC, Lordick F (2020). Gastric cancer. Lancet.

[2] Cao W, Chen HD, Yu YW, Li N, Chen WQ (2021). Changing profiles of cancer burden worldwide and in china: a secondary analysis of the global cancer statistics 2020. Chin Med J (Engl).

[3] Ma S, Zhou M, Xu Y (2023). Clinical application and detection techniques of liquid biopsy in gastric cancer. Mol Cancer.

[4] Bang YJ, Kim YW, Yang HK (2012). Adjuvant capecitabine and oxaliplatin for gastric cancer after d2 gastrectomy (CLASSIC): a phase 3 open-label, randomised controlled trial. Lancet.

[5] Tang XH, Guo T, Gao XY (2021). Exosome-derived noncoding RNAs in gastric cancer: functions and clinical applications. Mol Cancer.

[6] Namee NM, O'Driscoll L (1870). Extracellular vesicles and anti-cancer drug resistance. Biochim Biophys Acta Rev Cancer. 2018.

[7] Zhang Z, Wu H, Chong W, Shang L, Jing C, Li L (2022). Liquid biopsy in gastric cancer: predictive and prognostic biomarkers. Cell Death Dis.

[8] Qiu S, Xie L, Lu C (2022). Gastric cancer-derived exosomal mir-519a-3p promotes liver metastasis by inducing intrahepatic m2-like macrophage-mediated angiogenesis. J Exp Clin Cancer Res.

[9] Guo T, Tang XH, Gao XY (2022). A liquid biopsy signature of circulating exosome-derived mRNAs, miRNAs and lncRNAs predict therapeutic efficacy to neoadjuvant chemotherapy in patients with advanced gastric cancer. Mol Cancer.

[10] Zhang H, Deng T, Liu R (2017). Exosome-delivered EGFR regulates liver microenvironment to promote gastric cancer liver metastasis. Nat Commun.

[11] Sung H, Ferlay J, Siegel RL (2021). Global cancer statistics 2020: GLOBOCAN estimates of incidence and mortality worldwide for 36 cancers in 185 countries. CA Cancer J Clin.

[12] Plummer M, van Doorn LJ, Franceschi S (2007). Helicobacter pylori cytotoxin-associated genotype and gastric precancerous lesions. J Natl Cancer Inst.

[13] Na HK, Lee JY (2017). Molecular basis of alcohol-related gastric and colon cancer. Int J Mol Sci.

[14] Oliveira C, Pinheiro H, Figueiredo J, Seruca R, Carneiro F (2015). Familial gastric cancer: genetic susceptibility, pathology, and implications for management. Lancet Oncol.

[15] Lee YC, Chiang TH, Chou CK (2016). Association between helicobacter pylori eradication and gastric cancer incidence: a systematic review and meta-analysis. Gastroenterology.

[16] Park SH, Kang MJ, Yun EH, Jung KW (2022). Epidemiology of gastric cancer in korea: trends in incidence and survival based on korea central cancer registry data (1999-2019). J Gastric Cancer.

[17] Park Y, Ki M (2021). Population attributable fraction of helicobacter pylori infection-related gastric cancer in korea: a meta-analysis. Cancer Res Treat.

[18] Collatuzzo G, Pelucchi C, Negri E (2021). Exploring the interactions between helicobacter pylori (hp) infection and other risk factors of gastric cancer: a pooled analysis in the stomach cancer pooling (StoP) project. Int J Cancer.

[19] Chen YC, Malfertheiner P, Yu HT (2024). Global prevalence of helicobacter pylori infection and incidence of gastric cancer between 1980 and 2022. Gastroenterology.

[20] Bai Y, Xie T, Wang Z (2022). Efficacy and predictive biomarkers of immunotherapy in epstein-barr virus-associated gastric cancer. J Immunother Cancer.

[21] Pikula A, Kwietniewska M, Rawicz-Pruszynski K (2020). The importance of epstein-barr virus infection in the systemic treatment of patients with gastric cancer. Semin Oncol.

[22] Polakovicova I, Jerez S, Wichmann IA, Sandoval-Borquez A, Carrasco-Veliz N, Corvalan AH (2018). Role of microRNAs and exosomes in helicobacter pylori and epstein-barr virus associated gastric cancers. Front Microbiol.

[23] Kim SY, Kwak JH, Eun CS (2022). Gastric cancer risk was associated with dietary factors irritating the stomach wall: a case-control study in korea. Nutrients.

[24] Dabo B, Pelucchi C, Rota M (2022). The association between diabetes and gastric cancer: results from the stomach cancer pooling project consortium. Eur J Cancer Prev.

[25] Martimianaki G, Alicandro G, Pelucchi C (2022). Tea consumption and gastric cancer: a pooled analysis from the stomach cancer pooling (StoP) project consortium. Br J Cancer.

[26] Deng H, Gao J, Cao B (2023). LncRNA CCAT2 promotes malignant progression of metastatic gastric cancer through regulating CD44 alternative splicing. Cell Oncol (Dordr).

[27] Li H, Cao B, Zhao R (2022). CircDNMT1 promotes malignant progression of gastric cancer through targeting mir-576-3p/hypoxia inducible factor-1 alpha axis. Front Oncol.

[28] Mei Y, Feng X, Feng T (2022). Adjuvant chemotherapy in pt2n0m0 gastric cancer: findings from a retrospective study. Front Pharmacol.

[29] Mei Y, Wang S, Feng T (2021). Nomograms involving HER2 for predicting lymph node metastasis in early gastric cancer. Front Cell Dev Biol.

[30] Cho H, Yamada M, Sekine S (2021). Gastric cancer is highly prevalent in lynch syndrome patients with atrophic gastritis. Gastric Cancer.

[31] Zhang X, Li M, Chen S (2018). Endoscopic screening in asian countries is associated with reduced gastric cancer mortality: a meta-analysis and systematic review. Gastroenterology.

[32] Lyons K, Le LC, Pham YT (2019). Gastric cancer: epidemiology, biology, and prevention: a mini review. Eur J Cancer Prev.

[33] Patel TH, Cecchini M (2020). Targeted therapies in advanced gastric cancer. Curr Treat Options Oncol.

[34] Kalluri R, Lebleu VS (2020). The biology, function, and biomedical applications of exosomes. Science.

[35] Yanez-Mo M, Siljander PR, Andreu Z (2015). Biological properties of extracellular vesicles and their physiological functions. J Extracell Vesicles.

[36] Pegtel DM, Gould SJ (2019). Exosomes. Annu Rev Biochem.

[37] Kou R, Li T, Fu C (2024). Exosome-shuttled FTO from BM-MSCs contributes to cancer malignancy and chemoresistance in acute myeloid leukemia by inducing m6a-demethylation: a nano-based investigation. Environ Res.

[38] Li S, Dong R, Kang Z, Li H, Wu X, Li T (2023). Exosomes: another intercellular lipometabolic communication mediators in digestive system neoplasms?. Cytokine Growth Factor Rev.

[39] Guo W, Qiao T, Dong B, Li T, Liu Q, Xu X (2022). The effect of hypoxia-induced exosomes on anti-tumor immunity and its implication for immunotherapy. Front Immunol.

[40] Kimiz-Gebologlu I, Oncel SS (2022). Exosomes: large-scale production, isolation, drug loading efficiency, and biodistribution and uptake. J Control Release.

[41] Harding C, Heuser J, Stahl P (1983). Receptor-mediated endocytosis of transferrin and recycling of the transferrin receptor in rat reticulocytes. J Cell Biol.

[42] Pan BT, Johnstone RM (1983). Fate of the transferrin receptor during maturation of sheep reticulocytes in vitro: selective externalization of the receptor. Cell.

[43] Johnstone RM, Adam M, Hammond JR, Orr L, Turbide C (1987). Vesicle formation during reticulocyte maturation. Association of plasma membrane activities with released vesicles (exosomes). J Biol Chem.

[44] Raposo G, Nijman HW, Stoorvogel W (1996). B lymphocytes secrete antigen-presenting vesicles. J Exp Med.

[45] Valadi H, Ekstrom K, Bossios A, Sjostrand M, Lee JJ, Lotvall JO (2007). Exosome-mediated transfer of mRNAs and microRNAs is a novel mechanism of genetic exchange between cells. Nat Cell Biol.

[46] Zhang J, Li S, Li L (2015). Exosome and exosomal microRNA: trafficking, sorting, and function. Genomics Proteomics Bioinformatics.

[47] Mathieu M, Martin-Jaular L, Lavieu G, Thery C (2019). Specificities of secretion and uptake of exosomes and other extracellular vesicles for cell-to-cell communication. Nat Cell Biol.

[48] Hessvik NP, Llorente A (2018). Current knowledge on exosome biogenesis and release. Cell Mol Life Sci.

[49] Zhang H, Freitas D, Kim HS (2018). Identification of distinct nanoparticles and subsets of extracellular vesicles by asymmetric flow field-flow fractionation. Nat Cell Biol.

[50] Yang E, Wang X, Gong Z, Yu M, Wu H, Zhang D (2020). Exosome-mediated metabolic reprogramming: the emerging role in tumor microenvironment remodeling and its influence on cancer progression. Signal Transduct Target Ther.

[51] Jiang Y, Zhang H, Wang J, Liu Y, Luo T, Hua H (2022). Targeting extracellular matrix stiffness and mechanotransducers to improve cancer therapy. J Hematol Oncol.

[52] Chen X, Zhang S, Du K (2021). Gastric cancer-secreted exosomal x26nt increases angiogenesis and vascular permeability by targeting VE-cadherin. Cancer Sci.

[53] Wang J, Shen D, Li S (2023). LINC00665 activating wnt3a/beta-catenin signaling by bond with YBX1 promotes gastric cancer proliferation and metastasis. Cancer Gene Ther.

[54] Zhang Z, Sun C, Zheng Y, Gong Y (2022). CircFCHO2 promotes gastric cancer progression by activating the JAK1/STAT3 pathway via sponging mir-194-5p. Cell Cycle.

[55] Chen Z, Xie Y, Chen W, Li T, Chen X, Liu B (2021). RETRACTED: microRNA-6785-5p-loaded human umbilical cord mesenchymal stem cells-derived exosomes suppress angiogenesis and metastasis in gastric cancer via INHBA. Life Sci.

[56] Yoon JH, Choi BJ, Nam SW, Park WS (2022). Gastric cancer exosomes contribute to the field cancerization of gastric epithelial cells surrounding gastric cancer. Gastric Cancer.

[57] Shi H, Huang S, Qin M (2021). Exosomal circ_0088300 derived from cancer-associated fibroblasts acts as a mir-1305 sponge and promotes gastric carcinoma cell tumorigenesis. Front Cell Dev Biol.

[58] Wang Y, Shang K, Zhang N, Zhao J, Cao B (2021). Tumor-associated macrophage-derived exosomes promote the progression of gastric cancer by regulating the p38MAPK signaling pathway and the immune checkpoint PD-l1. Cancer Biother Radiopharm.

[59] Wei S, Peng L, Yang J (2020). Exosomal transfer of mir-15b-3p enhances tumorigenesis and malignant transformation through the DYNLT1/caspase-3/caspase-9 signaling pathway in gastric cancer. J Exp Clin Cancer Res.

[60] Zhu L, Zhang S, Chen S, Wu H, Jiang M, Liu A (2022). Exosomal mir-552-5p promotes tumorigenesis and disease progression via the PTEN/TOB1 axis in gastric cancer. J Cancer.

[61] Qu JL, Qu XJ, Zhao MF (2009). Gastric cancer exosomes promote tumour cell proliferation through PI3k/akt and MAPK/ERK activation. Dig Liver Dis.

[62] Arita T, Ichikawa D, Konishi H (2016). Tumor exosome-mediated promotion of adhesion to mesothelial cells in gastric cancer cells. Oncotarget.

[63] Miki Y, Yashiro M, Okuno T (2018). CD9-positive exosomes from cancer-associated fibroblasts stimulate the migration ability of scirrhous-type gastric cancer cells. Br J Cancer.

[64] Li R, Wang Y, Zhang X (2019). Exosome-mediated secretion of LOXL4 promotes hepatocellular carcinoma cell invasion and metastasis. Mol Cancer.

[65] Lin LY, Yang L, Zeng Q (2018). Tumor-originated exosomal lncUEGC1 as a circulating biomarker for early-stage gastric cancer. Mol Cancer.

[66] Shi Y, Wang Z, Zhu X (2020). Exosomal mir-1246 in serum as a potential biomarker for early diagnosis of gastric cancer. Int J Clin Oncol.

[67] Li W, Gao YQ (2018). MiR-217 is involved in the carcinogenesis of gastric cancer by down-regulating CDH1 expression. Kaohsiung J Med Sci.

[68] Ohshima K, Inoue K, Fujiwara A (2010). Let-7 microRNA family is selectively secreted into the extracellular environment via exosomes in a metastatic gastric cancer cell line. PLoS One.

[69] Greening DW, Gopal SK, Mathias RA (2015). Emerging roles of exosomes during epithelial-mesenchymal transition and cancer progression. Semin Cell Dev Biol.

[70] Shen X, Kong S, Ma S (2022). Hsa_circ_0000437 promotes pathogenesis of gastric cancer and lymph node metastasis. Oncogene.

[71] Pan L, Liang W, Fu M (2017). Exosomes-mediated transfer of long noncoding RNA ZFAS1 promotes gastric cancer progression. J Cancer Res Clin Oncol.

[72] Wu L, Zhang X, Zhang B (2016). Exosomes derived from gastric cancer cells activate NF-kappab pathway in macrophages to promote cancer progression. Tumour Biol.

[73] Gu J, Qian H, Shen L (2012). Gastric cancer exosomes trigger differentiation of umbilical cord derived mesenchymal stem cells to carcinoma-associated fibroblasts through TGF-beta/smad pathway. PLoS One.

[74] Yang H, Fu H, Wang B (2018). Exosomal mir-423-5p targets SUFU to promote cancer growth and metastasis and serves as a novel marker for gastric cancer. Mol Carcinog.

[75] Tokuhisa M, Ichikawa Y, Kosaka N (2015). Exosomal miRNAs from peritoneum lavage fluid as potential prognostic biomarkers of peritoneal metastasis in gastric cancer. PLoS One.

[76] Ohzawa H, Saito A, Kumagai Y (2020). Reduced expression of exosomal mir-29s in peritoneal fluid is a useful predictor of peritoneal recurrence after curative resection of gastric cancer with serosal involvement. Oncol Rep.

[77] Soeda N, Iinuma H, Suzuki Y (2019). Plasma exosome-encapsulated microRNA-21 and microRNA-92a are promising biomarkers for the prediction of peritoneal recurrence in patients with gastric cancer. Oncol Lett.

[78] Zhu M, Zhang N, Ma J, He S (2022). Integration of exosomal mir-106a and mesothelial cells facilitates gastric cancer peritoneal dissemination. Cell Signal.

[79] Ziegler KM, Considine RV, True E, Swartz-Basile DA, Pitt HA, Zyromski NJ (2016). Adipocytes enhance murine pancreatic cancer growth via a hepatocyte growth factor (HGF)-mediated mechanism. Int J Surg.

[80] Hoshino A, Costa-Silva B, Shen TL (2015). Tumour exosome integrins determine organotropic metastasis. Nature.

[81] Lobb RJ, Lima LG, Moller A (2017). Exosomes: key mediators of metastasis and pre-metastatic niche formation. Semin Cell Dev Biol.

[82] Mashouri L, Yousefi H, Aref AR, Ahadi AM, Molaei F, Alahari SK (2019). Exosomes: composition, biogenesis, and mechanisms in cancer metastasis and drug resistance. Mol Cancer.

[83] Qu JL, Qu XJ, Qu JL (2009). The role of cbl family of ubiquitin ligases in gastric cancer exosome-induced apoptosis of jurkat t cells. Acta Oncol.

[84] Liu D, Li C, Trojanowicz B (2016). CD97 promotion of gastric carcinoma lymphatic metastasis is exosome dependent. Gastric Cancer.

[85] Li C, Liu DR, Li GG (2015). CD97 promotes gastric cancer cell proliferation and invasion through exosome-mediated MAPK signaling pathway. World J Gastroenterol.

[86] Tanaka M, Kuriyama S, Itoh G (2017). Mesothelial cells create a novel tissue niche that facilitates gastric cancer invasion. Cancer Res.

[87] Wang F, Li B, Wei Y (2018). Tumor-derived exosomes induce PD1(+) macrophage population in human gastric cancer that promotes disease progression. Oncogenesis.

[88] Shen Y, Xue C, Li X (2019). Effects of gastric cancer cell-derived exosomes on the immune regulation of mesenchymal stem cells by the NF-kb signaling pathway. Stem Cells Dev.

[89] Zhang X, Shi H, Yuan X, Jiang P, Qian H, Xu W (2018). Tumor-derived exosomes induce n2 polarization of neutrophils to promote gastric cancer cell migration. Mol Cancer.

[90] Ji R, Zhang B, Zhang X (2015). Exosomes derived from human mesenchymal stem cells confer drug resistance in gastric cancer. Cell Cycle.

[91] Wang X, Zhang H, Bai M (2018). Exosomes serve as nanoparticles to deliver anti-mir-214 to reverse chemoresistance to cisplatin in gastric cancer. Mol Ther.

[92] Lampropoulou DI, Pliakou E, Aravantinos G, Filippou D, Gazouli M (2022). The role of exosomal non-coding RNAs in colorectal cancer drug resistance. Int J Mol Sci.

[93] Wang M, Qiu R, Yu S (2019). Paclitaxel-resistant gastric cancer MGC-803 cells promote epithelial-to-mesenchymal transition and chemoresistance in paclitaxel-sensitive cells via exosomal delivery of mir-155-5p. Int J Oncol.

[94] Yao W, Guo P, Mu Q, Wang Y (2021). Exosome-derived circ-PVT1 contributes to cisplatin resistance by regulating autophagy, invasion, and apoptosis via mir-30a-5p/YAP1 axis in gastric cancer cells. Cancer Biother Radiopharm.

[95] Lin H, Zhang L, Zhang C, Liu P (2020). Exosomal MiR-500a-3p promotes cisplatin resistance and stemness via negatively regulating FBXW7 in gastric cancer. J Cell Mol Med.

[96] Jingyue S, Xiao W, Juanmin Z, Wei L, Daoming L, Hong X (2019). TFAP2e methylation promotes 5-fluorouracil resistance via exosomal mir-106a-5p and mir-421 in gastric cancer MGC-803 cells. Mol Med Rep.

[97] Lin Z, Wu Y, Xu Y, Li G, Li Z, Liu T (2022). Mesenchymal stem cell-derived exosomes in cancer therapy resistance: recent advances and therapeutic potential. Mol Cancer.

[98] Xu Z, Zeng S, Gong Z, Yan Y (2020). Exosome-based immunotherapy: a promising approach for cancer treatment. Mol Cancer.

[99] Li X, Shao C, Shi Y, Han W (2018). Lessons learned from the blockade of immune checkpoints in cancer immunotherapy. J Hematol Oncol.

[100] Fan Y, Liu Y, Qu X (2019). ASO author reflections: the prognostic role of exosomal PD-l1 in patients with gastric cancer. Ann Surg Oncol.

[101] Fan Y, Che X, Qu J (2019). Exosomal PD-l1 retains immunosuppressive activity and is associated with gastric cancer prognosis. Ann Surg Oncol.

[102] Zhang M, Fan Y, Che X (2020). 5-FU-induced upregulation of exosomal PD-l1 causes immunosuppression in advanced gastric cancer patients. Front Oncol.

[103] Shao Y, Shen Y, Chen T, Xu F, Chen X, Zheng S (2016). The functions and clinical applications of tumor-derived exosomes. Oncotarget.

[104] Wee I, Syn N, Sethi G, Goh BC, Wang L (1871). Role of tumor-derived exosomes in cancer metastasis. Biochim Biophys Acta Rev Cancer. 2019.

[105] Ocansey D, Zhang L, Wang Y (2020). Exosome-mediated effects and applications in inflammatory bowel disease. Biol Rev Camb Philos Soc.

[106] Zhao G, Zhou A, Li X (2021). The significance of exosomal RNAs in the development, diagnosis, and treatment of gastric cancer. Genes (Basel).

[107] Zhu L, Sun HT, Wang S (2020). Isolation and characterization of exosomes for cancer research. J Hematol Oncol.

[108] Huang Z, Zhu D, Wu L (2017). Six serum-based miRNAs as potential diagnostic biomarkers for gastric cancer. Cancer Epidemiol Biomarkers Prev.

[109] Imamura T, Komatsu S, Ichikawa D (2017). Low plasma levels of mir-101 are associated with tumor progression in gastric cancer. Oncotarget.

[110] Kumata Y, Iinuma H, Suzuki Y (2018). Exosome-encapsulated microRNA-23b as a minimally invasive liquid biomarker for the prediction of recurrence and prognosis of gastric cancer patients in each tumor stage. Oncol Rep.

[111] Yun J, Han SB, Kim HJ (2019). Exosomal mir-181b-5p downregulation in ascites serves as a potential diagnostic biomarker for gastric cancer-associated malignant ascites. J Gastric Cancer.

[112] Song Q, Lv X, Ru Y (2022). Circulating exosomal gastric cancer-associated long noncoding RNA1 as a noninvasive biomarker for predicting chemotherapy response and prognosis of advanced gastric cancer: a multi-cohort, multi-phase study. EBioMedicine.

[113] Zheng P, Zhang H, Gao H (2020). Plasma exosomal long noncoding RNA lnc-SLC2a12-10:1 as a novel diagnostic biomarker for gastric cancer. Onco Targets Ther.

[114] Zheng P, Gao H, Xie X, Lu P (2022). Plasma exosomal hsa_circ_0015286 as a potential diagnostic and prognostic biomarker for gastric cancer. Pathol Oncol Res.

[115] Wang H, Zeng X, Zheng Y, Wang Y, Zhou Y (2021). Exosomal circRNA in digestive system tumors: the main player or coadjuvants?. Front Oncol.

[116] Tang G, Wang J, Dong W, Dai K, Du J (2022). Exosomal miRNA expression profiling and the roles of exosomal mir-4741, mir-32, mir-3149, and mir-6727 on gastric cancer progression. Biomed Res Int.

[117] Zhang Y, Han T, Feng D (2020). Screening of non-invasive miRNA biomarker candidates for metastasis of gastric cancer by small RNA sequencing of plasma exosomes. Carcinogenesis.

[118] Zheng GD, Xu ZY, Hu C (2021). Exosomal mir-590-5p in serum as a biomarker for the diagnosis and prognosis of gastric cancer. Front Mol Biosci.

[119] Liu X, Chu KM (2020). Exosomal miRNAs as circulating biomarkers for prediction of development of haematogenous metastasis after surgery for stage II/III gastric cancer. J Cell Mol Med.

[120] Ge L, Zhang N, Li D, Wu Y, Wang H, Wang J (2020). Circulating exosomal small RNAs are promising non-invasive diagnostic biomarkers for gastric cancer. J Cell Mol Med.

[121] Zhou Y, Li R (2024). Exosomal mir-502-5p suppresses the progression of gastric cancer by repressing angiogenesis through the wnt/beta-catenin pathway. Ir J Med Sci.

[122] Xiao H, Fu J, Liu R, Yan L, Zhou Z, Yuan J (2024). Gastric cancer cell-derived exosomal mir-541-5p induces m2 macrophage polarization through DUSP3/JAK2/STAT3 pathway. BMC Cancer.

[123] Zhou H, Shen W, Zou H, Lv Q, Shao P (2020). Circulating exosomal long non-coding RNA h19 as a potential novel diagnostic and prognostic biomarker for gastric cancer. J Int Med Res.

[124] Xu H, Zhou J, Tang J (2020). Identification of serum exosomal lncRNA MIAT as a novel diagnostic and prognostic biomarker for gastric cancer. J Clin Lab Anal.

[125] Li S, Zhang M, Zhang H (2020). Exosomal long noncoding RNA lnc-GNAQ-6:1 may serve as a diagnostic marker for gastric cancer. Clin Chim Acta.

[126] Cai C, Zhang H, Zhu Y (2019). Serum exosomal long noncoding RNA pcsk2-2:1 as a potential novel diagnostic biomarker for gastric cancer. Onco Targets Ther.

[127] Piao HY, Guo S, Wang Y, Zhang J (2020). Exosomal long non-coding RNA CEBPA-as1 inhibits tumor apoptosis and functions as a non-invasive biomarker for diagnosis of gastric cancer. Onco Targets Ther.

[128] Zhao R, Zhang Y, Zhang X (2018). Exosomal long noncoding RNA HOTTIP as potential novel diagnostic and prognostic biomarker test for gastric cancer. Mol Cancer.

[129] Li Q, Shao Y, Zhang X (2015). Plasma long noncoding RNA protected by exosomes as a potential stable biomarker for gastric cancer. Tumour Biol.

[130] Yoon JH, Byun HJ, Kim SY, Jung DH, Lee SK (2024). Exosomal LINC00853 promotes progression of gastric cancer via the MAP17/PDZK1/AKT signaling pathway. Noncoding RNA Res.

[131] Shao Y, Tao X, Lu R (2020). Hsa_circ_0065149 is an indicator for early gastric cancer screening and prognosis prediction. Pathol Oncol Res.

[132] Yoon JH, Ham IH, Kim O (2018). Gastrokine 1 protein is a potential theragnostic target for gastric cancer. Gastric Cancer.

[133] Ren W, Zhang X, Li W (2019). Exosomal miRNA-107 induces myeloid-derived suppressor cell expansion in gastric cancer. Cancer Manag Res.

[134] Xue X, Huang J, Yu K (2020). YB-1 transferred by gastric cancer exosomes promotes angiogenesis via enhancing the expression of angiogenic factors in vascular endothelial cells. BMC Cancer.

[135] Fu H, Yang H, Zhang X (2018). Exosomal TRIM3 is a novel marker and therapy target for gastric cancer. J Exp Clin Cancer Res.

[136] Zhu L, Li J, Gong Y (2019). Exosomal tRNA-derived small RNA as a promising biomarker for cancer diagnosis. Mol Cancer.

[137] Zhang Y, Cai F, Liu J (2018). Transfer RNA-derived fragments as potential exosome tRNA-derived fragment biomarkers for osteoporosis. Int J Rheum Dis.

[138] Skryabin GO, Beliaeva AA, Enikeev AD, Tchevkina EM (2024). Extracellular vesicle miRNAs in diagnostics of gastric cancer. Biochemistry (Mosc).

[139] Shaker F, Razi S, Rezaei N (2024). Circulating miRNA and circulating tumor DNA application as liquid biopsy markers in gastric cancer. Clin Biochem.

[140] Cai ZR, Zheng YQ, Hu Y Construction of exosome non-coding RNA feature for non-invasive, early detection of gastric cancer patients by machine learning: a multi-cohort study. Gut. 2025: gutjnl-2024-333522.

[141] Peng H, Ji W, Zhao R (2020). Exosome: a significant nano-scale drug delivery carrier. J Mater Chem B.

[142] Zhao X, Wu D, Ma X, Wang J, Hou W, Zhang W (2020). Exosomes as drug carriers for cancer therapy and challenges regarding exosome uptake. Biomed Pharmacother.

[143] Liu X, Lu Y, Xu Y (2019). Exosomal transfer of mir-501 confers doxorubicin resistance and tumorigenesis via targeting of BLID in gastric cancer. Cancer Lett.

[144] Zheng P, Chen L, Yuan X (2017). Exosomal transfer of tumor-associated macrophage-derived mir-21 confers cisplatin resistance in gastric cancer cells. J Exp Clin Cancer Res.

[145] Usman WM, Pham TC, Kwok YY (2018). Efficient RNA drug delivery using red blood cell extracellular vesicles. Nat Commun.

[146] Zhang H, Wang Y, Bai M (2018). Exosomes serve as nanoparticles to suppress tumor growth and angiogenesis in gastric cancer by delivering hepatocyte growth factor siRNA. Cancer Sci.

[147] Zhou J, Tan X, Tan Y, Li Q, Ma J, Wang G (2018). Mesenchymal stem cell derived exosomes in cancer progression, metastasis and drug delivery: a comprehensive review. J Cancer.

[148] Rivoltini L, Chiodoni C, Squarcina P (2016). TNF-related apoptosis-inducing ligand (TRAIL)-armed exosomes deliver proapoptotic signals to tumor site. Clin Cancer Res.

[149] Barok M, Puhka M, Vereb G, Szollosi J, Isola J, Joensuu H (2018). Cancer-derived exosomes from HER2-positive cancer cells carry trastuzumab-emtansine into cancer cells leading to growth inhibition and caspase activation. BMC Cancer.

[150] Peng J, Wu Y, Deng S (2023). Effect of the application of exosome on gastric cancer. Comb Chem High Throughput Screen.

[151] Li Q, Wang D, Ding D (2021). The role and application of exosomes in gastric and colorectal cancer. Front Pharmacol.

